# Supplementary material for: Echo chambers can emerge without algorithmic personalization or a preference for homogeneity
Source: PLoS One. 2026 May 6;21(5):e0347207. doi: 10.1371/journal.pone.0347207 (PMC13148672; doi:10.1371/journal.pone.0347207)
Supplement: Attachment — Submitted filename: reviewerresponse.docx [file pone.0347207.s001.docx]

**Dear Editor and Reviewers,**

We would like to thank you for the opportunity to revise our manuscript and for the insightful and constructive feedback provided during the review process. We are grateful to the reviewers for their thoughtful comments, which have helped us clarify the presentation, strengthen the analysis, and improve the overall contribution of the paper. We have carefully considered all suggestions and have revised the manuscript accordingly. Below, we provide a detailed, point-by-point response to each comment.

**Reviewer #1:** The paper presents an interesting toy model to represent ideologically aligned communities in social networks. The manuscript is well-written and easy to read. The results are interesting and support the discussions. However, I believe that there are some points that need improvement before publication. In the following, I present my concerns in detail.

**Author:** We thank Reviewer #1 for their thoughtful and constructive feedback. We appreciate the positive assessment of the model’s contribution, the clarity of the manuscript, and the relevance of the results. The reviewer’s comments were extremely helpful in identifying areas where the presentation and analysis could be strengthened. We have revised the manuscript accordingly and provide detailed responses to each point below.

**Reviewer:** 1 - On page 4, second line, the author argues that in online social networks, "interaction rarely follows spatial logics". However, people often connect with their offline friends on social media. Consequently, it is not rare to follow spatial logics. For instance, Laniado et al. argue that "friendship ties belonging to denser connected groups tend to arise at shorter spatial distances than social ties established between members belonging to different groups."
See this reference: Laniado, D., Volkovich, Y., Scellato, S., Mascolo, C., & Kaltenbrunner, A. (2018). The impact of geographic distance on online social interactions. Information Systems Frontiers, 20(6), 1203-1218.

**Author:** We thank the reviewer for pointing this out. We agree that this was poorly formulated in the original version, as offline spatial proximity indeed is central to shaping online ties. Our intention was not to deny this, but to highlight that many online interactions are organized via bounded groups and communities rather than geographic neighborhoods, which leads to different interaction structures, in turn shaping online phenomena. While networks and groups are to certain degree shaped by spatial context, the dynamics of leaving and entering online communities differs from the dynamics of moving between geographical areas. We have now revised the text to clarify this claim and to explicitly acknowledge work documenting spatial structure in online networks (e.g., Laniado et al. 2018).

**Reviewer:** 2 - The results section starts without describing the parameters used. Therefore, it took me some time to understand the first result. I suggest just adding these parameters to the first paragraph (N=100, C=20, k=10).

**Author**: We agree and now explicitly state the parameter values used in the first set of simulations at the beginning of the Results section.

**Reviewer:**3 - Each result is shown for a single (or a few) execution(s) of the dynamics. So, it is not possible to guarantee that the result will always be the same. Performing the experiments more times would make the results more reliable and robust.

4 - Some of the parameters could be better explored (or justified). Why only N=100 and C=20? I suggest exploring these parameters and testing higher values of N.

**Author:** We thank the reviewer for this suggestion. We have now rerun the model with 30 instead of 10 runs per step in Fig 2 and Fig 4. As before, the figures are reported with 0.95 confidence intervals.

We have also revised the model parameters, to make them more intuitive. First, in the original manuscript, N denoted the number of agents in *each community*, not the overall number of agents, meaning that the total number of agents was N*C=2,000. This was however both unintuitive, and far from clear in the manuscript text. We have therefore revised the model so that N denotes the total number of agents, not the number of agents per community. Second, we have redefined timesteps, so that each timestep represents sweep over each agent, instead of an individual move. This is standard procedure in ABMs, and makes the model more intuitive, and comparable across values of N.

We have also clarified in the text and figure captions that summary statistics (e.g. segregation indices) are averaged over multiple independent runs, and we have increased this to 30 runs per each combination of parameters.

In addition, we have conducted robustness checks varying the number of agents 𝑁 and communities *C* and confirmed that the qualitative tipping pattern is unchanged. These are now reported in Figure 3.

**Reviewer:** 5 - In the title of Figure 4, there is a variable noise. However, I did not understand its meaning.

**Author**: We apologize for this error – this was a left-over reference from a previous version of the model. The title of the plot has been revised.

**Reviewer:** 6 - I found the first paragraph of Section 3.1 a bit confusing. It says: "We introduce a parameter b controlling the proportion of interaction partners who are artificially selected to share the focal agent's opinion."
If I understand correctly, the filter bubble version does not alter the opinions of the agents for subsequent iterations of the model; instead, it modifies the value of "f of interactors". Am I correct? I suggest the author rewrite this paragraph to clarify this information.

**Author**: We agree that this point was not sufficiently clear. In the revised manuscript we now state explicitly that the filter-bubble mechanism only affects which interaction partners are sampled for the focal agent; it does not change any agent’s underlying opinion or the actual composition of communities.

We again thank the reviewer for their constructive and in-depth comments.

**Reviewer #2:**

**Reviewer**: In this paper the author extends the Schelling model with a simple modification: agents, instead of being located in a geometrical space are located within C abstract communities and can relocate to any other community, without spatial or network constraints. The author shows how this decreases the segregation threshold from 0.3 to 0.1 roughly. Then the author shows how the introduction of a filter bubble effect may actually reduce the segregation (perceived and effective). These results are demonstrated analytically and numerically.

The article is well written and structured, relevant literature was cited, the introduction and the results are clear. The study seems sound and robust although a methodology section describing the model and the analytical solution and the metrics used would be of help. The results are interesting and contribute to a timely and ongoing discussion with a well put perspective. The code is publicly available.

**Author: We thank Reviewer #2 for their positive and constructive assessment of the manuscript.** We are grateful for the encouraging remarks regarding the clarity of the paper, the relevance of the results, and the contribution to ongoing debates on online segregation. We also appreciate the reviewer’s helpful suggestions—particularly the request for a clearer methodological section detailing the model, analytical derivations, and metrics.

We have revised the manuscript accordingly and provide detailed responses to each point below.

**Reviewer:** However, there are a number of limitations that need to be addressed When the author claims that the segregation is not a result of individual choice, what does this mean? There is still a user evaluating their environment and deciding to relocate. Maybe the author could reformulate such claims and just state that there is no intentional homophilic dynamics guiding segregation. Users are not looking for alike individuals, they're just moving away from different ones.

**Author**: We thank the reviewer for this clarification. Our intention is indeed not to deny that agents make individual relocation decisions, but to highlight that high levels of segregation arises even with very low levels of homophilic behavior. We have revised the wording throughout the paper to emphasize that the model captures segregation emerging in the absence of explicit preferences for like-minded others or algorithmic targeting, rather than in the absence of individual decision-making per se.

**Reviewer:** One aspect that I find conceptually unclear is the way the model is set up. We have C communities, and users who can switch communities randomly. While I understand that the goal is to show that segregation can emerge even without intentional behavior, the assumption that users change communities at random is rather strong and somewhat puzzling.

**Author:** We agree that random relocation is a strong simplifying assumption. We introduce it deliberately to isolate a minimal mechanism: even when agents have no information about alternative communities and do not seek out like-minded others, simple dissatisfaction-based exit is sufficient to generate segregation. This mirrors the logic of Schelling’s original model, where dissatisfied agents likewise relocate to randomly selected locations. It also follows the modelling tradition in statistical physics, where random movement is treated as the lowest-information baseline.

Importantly, this makes the result stronger: if segregation emerges even in the scenario where one would least expect it—when agents move blindly and without any homophilic intent—then more realistic relocation rules (e.g., preferential movement toward communities with more congenial compositions) would only amplify the effect.

We have now clarified this rationale in the revised manuscript and explicitly note that the model identifies a conservative lower bound on segregation dynamics.

**Reviewer:** The introduction of the filter-bubble mechanism raises additional questions. As I understand it, users are more likely to see opinions similar to their own within their current community. Yet this seems to reduce segregation, because if users are less exposed to disagreeing opinions, they have fewer incentives to change communities. This could be coherent with some interpretations, but it is not entirely convincing. If the communities are meant to represent real-world communities, for instance, Reddit subreddits, I am not sure such an internal filter-bubble mechanism exists. Once a user is in a subreddit, content is typically shown quite uniformly (subject to popularity), without strong personalization within the community itself. If anything, filter-bubble effects might influence which community a user enters in the first place, not what they see after joining.
Of course, the interpretation would be different if “communities” are intended to represent sets of nodes in a network that are highly connected internally and weakly connected externally. In that case, a network-based model might be more appropriate, or at least this interpretation should be made explicit. Overall, I find it difficult to grasp the intended meaning of the environment and of the filter-bubble mechanism as currently described. Clearer justification, ideally supported by empirical examples, would help substantially.

**Author**: We thank the reviewer for raising this important point about the realism of the filter-bubble mechanism. While Reddit is indeed often described as a community-driven platform with largely uniform content exposure within a subreddit, Reddit now uses personalized ranking both on the home feed and *within* subreddits, based on individual engagement patterns such as past clicks, votes, dwell time, and scrolling behavior. As a result, different users of the *same* subreddit may be shown different subsets of posts from the same content pool.

In this light, the mechanism we model should be interpreted not as altering the *membership* or *composition* of a subreddit, but as an abstraction of how ranking algorithms shape which posts a user encounters. Since users typically see only a very small fraction of the content posted in large subreddits, ranking systems effectively determine the subset of opinions a user interacts with. Even modest personalization – such as surfacing posts that align with a user’s previous engagement – can increase the likelihood that the interaction sample is skewed toward like-minded content, even if the underlying community remains ideologically diverse.

We have revised the manuscript to clarify this interpretation: the filter-bubble parameter models algorithmic ranking of visible posts, not changes in the underlying opinions or community structure. This aligns the model with empirical realities of major platforms (including Reddit, YouTube, Facebook, TikTok), where personalization affects *exposure* rather than *group membership*. The goal is not to reproduce the exact technicalities of any single platform but to capture the general mechanism through which curated exposure reduces dissatisfaction and, consequently, the likelihood of exit, thereby stabilizing mixed communities.

**Reviewer:** A related point concerns the description of the resulting dynamics as “surprising” or “counterintuitive.” In fact, the outcome seems quite intuitive: if relocation is driven by exposure to disagreeing opinions, and the algorithm reduces the probability of seeing disagreeing opinions, then it naturally slows down segregation dynamics. This logic has also been discussed in previous work on opinion dynamics on adaptive networks (e.g., Pansanella et al. 2022, Modeling algorithmic bias: simplicial complexes and evolving network topologies, Applied Network Science; for a similar discussion also Valensise, Carlo M., Matteo Cinelli, and Walter Quattrociocchi. "The drivers of online polarization: Fitting models to data." Information Sciences 642 (2023): 119152; maybe also Cinus, Federico, et al. "The effect of people recommenders on echo chambers and polarization." Proceedings of the International AAAI Conference on Web and Social Media. Vol. 16. 2022.).

**Author**: We thank the reviewer for this helpful comment. We agree that, at a high level, the mechanism the reviewer describes is intuitive: if relocation is triggered by exposure to disagreeing content, then any algorithmic reduction in such exposure will mechanically slow segregation dynamics. We now make this logic explicit in the text.

At the same time, our model differs from prior work in a way that makes the outcome less straightforward ex ante. In many opinion-dynamics and adaptive-network models (including Pansanella et al. 2022; Valensise et al. 2023), reducing exposure to disagreeing opinions tends to *reinforce* homophily or fragmentation, because content filtering typically amplifies existing similarity rather than increasing cross-cutting exposure. By contrast, in our model filter-bubble strength interacts with the relocation mechanism, not with opinion change or network rewiring. As a result, stronger filtering reduces relocation pressure but does not increase homophilous link formation. The combination of these two forces produces a parameter region in which higher levels of algorithmic filtering *decrease* segregation and lead to more diverse local environments. While this outcome follows from the logic of our model once spelled out, it is not the standard expectation from the broader filter-bubble literature, where filtering is typically associated with *increased* homogeneity.

We now clarify this distinction in the manuscript and add references to Pansanella et al. (2022), Valensise et al. (2023), and Cinus et al. (2022), noting where our mechanism aligns with and where it diverges from their assumptions. We also soften the language describing the effect as “surprising” and instead emphasize that it emerges from the specific interaction between algorithmic filtering and relocation in our framework.

**Reviewer:** More generally, I would suggest tempering some of the stronger claims. The model is ultimately a stylized toy model, and we have very limited knowledge of how real platforms actually operate. Making this explicit would, in my view, strengthen the paper. Suggesting that the results explain the segregation found in online environment is an overstatement. First of all the results are not directly compared to any dataset or empirical measure of segregation from different platforms. Second, there are plenty of works in literature that claim to have explained polarization/echo chamber/segregation with their model that are all equally valid and sound.

**Author:** We fully agree that our model is a stylized toy model and that we do not claim to fully “explain” observed segregation on real platforms. We have revised the paper to make this limitation explicit, emphasizing that the model identifies a plausible mechanism rather than a complete explanation, and that it should be seen as complementing existing modeling literature. We have added a paragraph in the conclusion highlighting the limitations of the model, and that it should be interpreted with care.

**Reviewer:** I would suggest some degree of empirical validation to make the claims more robust using dataset from different social media platforms, if possible, otherwise a justification on why it is not possible/does not make sense.

**Author**: We thank the reviewer for this excellent suggestion. In the revised manuscript we have now added an empirical analysis to support the model’s core behavioral mechanism using a large-scale longitudinal dataset from Reddit, focusing specifically on the subreddit r/MensRights: a famous example of an online echo chamber. The full comment history is available, allowing us to reconstruct the month-by-month evolution of each user’s linguistic position and participation trajectory.

Our analysis proceeds in two steps. First, we embed each user’s monthly linguistic activity using sentence-transformer embeddings and compute their semantic distance from the community’s monthly centroid. Second, we model whether users exit the community in subsequent months as a function of this distance. The results show a robust and monotonic relationship: users who are further from the community’s semantic center are significantly more likely to leave the subreddit. A one-standard-deviation increase in distance is associated with a 16–18% increase in the odds of exit, and exit probabilities rise from 13.5% in the closest quartile of users to 20% in the most distant quartile. This pattern closely matches the selective-retention mechanism in our agent-based model, where divergence from local environments generates relocation cascades and, ultimately, segregation. We now report these findings in the manuscript and explicitly discuss how they reinforce the theoretical contribution.

Regarding the reviewer’s suggestion to extend the analysis across additional platforms: our mechanism is platform-general and concerns the structural consequences of group-based interaction architectures rather than platform-specific affordances. However, applying our validation approach beyond Reddit is currently limited by data accessibility. Unlike Reddit, most major platforms (e.g., Facebook, Instagram, TikTok, and contemporary Twitter/X) do not provide public, user-level historical data at the granularity required to reconstruct full participation trajectories and compute semantic drift over time. In contrast, Reddit remains one of the only environments where complete longitudinal comment histories are openly available at scale. We have therefore chosen a platform where the empirical data permits an appropriate and methodologically rigorous test of the model’s predictions.

We hope that the new empirical section addresses the reviewer’s request for validation and strengthens the contribution of the paper.
