# Supplementary material for: Echo chambers can emerge without algorithmic personalization or a preference for homogeneity
Source: PLoS One. 2026 May 6;21(5):e0347207. doi: 10.1371/journal.pone.0347207 (PMC13148672; doi:10.1371/journal.pone.0347207)
Supplement: Attachment — Submitted filename: Response to Reviewers.docx [file pone.0347207.s002.docx]

**Response to Reviewers**

Dear Editor,

Thank you for the opportunity to revise this manuscript. I am grateful for the careful reading and constructive guidance from the editor and both reviewers. I have revised the manuscript substantially in response to these comments. The main changes focus on clarifying the framing of the model, avoiding language that could imply stronger causal claims than warranted, and narrowing the interpretation of the empirical section. In particular, I now distinguish more clearly between homophily in the broader literature and the weaker preference structure encoded in the model; I replace references to “algorithmic design” with the more precise term “algorithmic personalization”; I define the paper’s contribution in terms of exit dynamics in bounded online communities rather than broad structural claims about platforms in general; and I recast the empirical analysis explicitly as an illustrative plausibility check of a single micro-level assumption rather than as validation of the model’s full macro-dynamics. I have also expanded the discussion of limitations, standardized terminology, and addressed the reviewer’s specific textual suggestions.

In relation to the editor’s comments, I have carried out the following changes:

First, I have clarified the framing of the model so that it no longer suggests that preferences are absent. Instead, the revised manuscript now consistently states that the model does not require strong homophily, strong preferences for homogeneous environments, or active search for like-minded groups. It now emphasizes that the model includes only a weak dissatisfaction threshold under conditions of strong disagreement, rather than a preference for diversity or an absence of preference altogether.

Second, I have revised the manuscript to reduce language that could be read as making causal claims beyond what the evidence supports. In particular, I have removed or softened formulations that implied that the empirical section validates the model’s core dynamics. The empirical analysis is now consistently framed as an illustrative plausibility check of one micro-level assumption: that users whose language is more distant from a community’s monthly centroid are more likely to exit.

Third, I have expanded the discussion of limitations, especially in the empirical section. The revised manuscript now explicitly discusses unobserved user-level heterogeneity, the fact that centroid distance may reflect topic choice or rhetorical style rather than ideology alone, the behavioral ambiguity of exit, and the lack of conditional independence in the logistic regression because users contribute repeated observations and exit is an absorbing state.

Finally, I revised the title, abstract, introduction, empirical section, and discussion to make the paper’s contribution more precise and more modest where appropriate, while preserving its core argument.

Below I respond point by point.

**Response to Reviewer #1**

**Comment:**
The author has answered all my questions and significantly improved the overall quality of the paper.

**Response:**
Thank you for the careful reading and constructive feedback across both rounds. I appreciate the helpful suggestions, which improved the clarity and precision of the manuscript.

**Response to Reviewer #2**

**Comment**:
The sentence in the abstract “Taken together, these findings recast online echo chambers not as a result of human preferences or algorithmic design, but as a structural consequence of how online platforms reorganize social interaction,” is conceptually unclear and, as currently phrased, misleading. I interpreted it as “Even in the absence of explicit homophily or personalized recommendation, the basic organizational features of online platforms (bounded communities, easy exit, aggregation of interactions) can generate echo chambers as an emergent outcome”, but this is just an interpretation. However, as I already stated in the first round of review, human preference is still present - even if it is not strong - since exiting due to disagreement and the level of tolerated disagreement are human preferences. In principle, also saying that the dynamic is not a result of “algorithmic design” is not entirely correct: every mechanism related to an online platform is due to algorithmic design, but I think that here one can just change design into personalization, and the meaning becomes clearer. The notion of a “structural consequence” is not defined, and it is not clear what is meant by platforms “reorganizing social interaction”, especially given that the model does not include any active platform intervention. I recommend rephrasing the sentence in a less ambiguous way, stating what the findings suggest, using well-defined terms, and avoiding overstating, attributing agency, or causal mechanisms. The comments on this sentence should be taken as an example to guide revision of the whole text, being careful with terminology and avoiding ambiguous terms that may suggest causality or overstate effects.

**Response**:
Thank you. I agree with this concern and have revised the abstract and the manuscript more broadly accordingly.

The abstract has been rewritten to avoid the earlier phrasing. It now states that echo chambers need not depend on users seeking *homogeneous* environments or on algorithmic personalization alone, but can also emerge from exit dynamics in bounded online communities. This formulation is more precise in three respects. It no longer implies that preferences are absent; it replaces “algorithmic design” with the narrower and more appropriate term “algorithmic personalization”; and it specifies the mechanism in terms of bounded communities and exit dynamics rather than undefined structural language. I have also shifted from “homophily” to “preference for homogeneity”, as this is more accurate.

I revised the paper as a whole to follow the same principle. In particular, I removed or rephrased statements suggesting that the model excludes human preference entirely, and I no longer describe the mechanism as replacing user preference or platform design accounts. Instead, the manuscript now presents the argument as identifying an additional mechanism through which segregation can emerge, alongside the mechanisms emphasized in the existing literature.

More generally, I revised terminology throughout to reduce ambiguity and avoid overstating the claims. For example, I replaced several broad references to “platform design” or “reorganizing social interaction” with more specific descriptions of bounded communities, limited local interaction, and easy exit.

**Comment**:
The newly added empirical analysis on r/MensRights is a useful addition, but its contribution should be framed more modestly. The logistic regression shows that users whose language is farther from the monthly community centroid are more likely to cease participation in the subsequent time window. This is an interesting and plausible descriptive finding, and it is broadly consistent with the model's micro-level assumption. However, the analysis does not, and cannot, provide evidence for the broader Schelling-type dynamics emphasized in the theoretical sections.

**Response**:
Thank you. I have revised the empirical section to frame it more modestly and more precisely.

Most importantly, I now describe the empirical section consistently as an illustrative plausibility check of a single micro-level assumption rather than as validation of the model’s broader dynamics. The introduction, the empirical section itself, and the discussion all now use this narrower framing. I removed language suggesting that the analysis demonstrates or supports the model’s macro-level Schelling-type dynamics.

I also revised the wording in the results and interpretation so that the section now states that the findings are compatible with the model’s core micro-level assumption, namely that users who are more distant from a community’s evolving center are more likely to exit.

**Comment**:
Several limitations deserve more explicit discussion:

- The regression does not control for unobserved user-level heterogeneity (e.g., stable differences in engagement style or propensity to churn), as no user fixed effects or random effects are included. As a result, the estimated association between linguistic distance and exit may reflect selection effects rather than dissatisfaction with minority status.
- Most importantly, linguistic distance from the centroid conflates ideological disagreement with other forms of divergence, such as topic choice or rhetorical style, and is itself endogenously shaped by participation and exit.
- Exit is behaviorally ambiguous and may reflect factors unrelated to dissatisfaction with community composition (e.g., reduced platform use or migration elsewhere).
- The observations are not independent, as users contribute multiple observations and distances are jointly determined through a shared centroid. Because observations are repeated over time for the same users and exit is an absorbing state, the conditional independence assumption of logistic regression is violated.

**Response**:
Thank you. I have added an explicit limitations paragraph to the empirical section that addresses each of these points directly.

First, the revised manuscript now states that the regression does not include user fixed effects or random effects, and that the observed association may therefore partly reflect stable differences in users’ engagement style or propensity to exit.

Second, the manuscript now clarifies that centroid distance should not be interpreted as a direct measure of ideology. I revised the section to shift from “ideological distance” to “linguistic distance” or “semantic distance,” and I now explicitly state that distance from the centroid may reflect ideological disagreement, topic choice, rhetorical style, or other forms of divergence. I also note that this measure is endogenously shaped by participation and exit patterns.

Third, I now note explicitly that exit is behaviorally ambiguous and may reflect reduced platform use, migration to other communities, or other factors unrelated to dissatisfaction with community composition.

Fourth, I added explicit discussion of the dependence structure in the data: users contribute repeated observations over time, exit is an absorbing state, and distances are jointly determined through a shared monthly centroid. The manuscript now states that these features violate the conditional independence assumption of standard logistic regression and may affect the precision of the estimated coefficients.

**Comment**:
While the authors do note that the results are “descriptive rather than causal,” some passages nonetheless suggest stronger empirical support for the model’s mechanism than the analysis can justify. I recommend consistently framing this section as an illustrative plausibility check of a single micro-level assumption, rather than as empirical validation of the model’s core dynamics.

**Response**:
I agree with this point and have revised the manuscript accordingly. The empirical section is now consistently framed as an illustrative plausibility check of a single micro-level assumption. I removed formulations suggesting that the empirical results validate the model’s broader mechanism or macro-level dynamics. This change has been made in the abstract, introduction, empirical section, and discussion, so that the framing is now consistent throughout.

**Comment**:
In the abstract: “as an structural consequence” → as a structural consequence

**Response**:
Corrected. The abstract was rewritten more extensively, and this grammatical error was removed.

**Comment**:
“randomly distributed randomly across C communities”: remove redundancies

**Response**:
Corrected. This phrase now reads “distributed uniformly at random across C discrete communities.”

**Comment**:
Inconsistent use of hyphenation: “micro level”, “macro level” vs. “micro-level”, “macro-level” → standardize to hyphenated forms throughout

**Response**:
Thank you. I checked for such errors throughout the manuscript, and they have all been hyphenated consistently. (Note, however, that this may have been the result of an issue with the submission system, as I could not find any non-hyphenated versions of these terms in the previous version of the manuscript. I can therefore not guarantee that the version which reaches the reviewer will be without such errors. If this is the case, I do apologize, and will work with the journal to address the issue.)

**Comment**:
Words such as “sharp,” “striking,” “dramatic,” “paradoxical,” and “counterintuitive” are sometimes overused. While acceptable in moderation, I suggest reducing repetition, especially in the Results and Discussion sections.

**Response**:
I revised the Results and Discussion to reduce the repetition of these terms. In several places I replaced them with more neutral language such as “clear,” “nonlinear,” “substantial,” or “notable,” and in other places I simply removed the intensifying adjective altogether.

**Comment**:
Terminology consistency: “exit,” “disengage,” and “leave” are used somewhat interchangeably. I recommend choosing one primary term (e.g., exit) and using others only when stylistically necessary.

**Response**:
Thank you. I adopted “exit” as the primary term throughout the manuscript and revised a number of passages to improve consistency. Other terms are now used only sparingly where needed for style or readability.

**Comment**:
Avoid causal language. Even small phrases such as “predicts exit” could be replaced, for example, with “is associated with exit” for consistency with the stated descriptive scope.

**Response**:
I revised the empirical section throughout to reduce causal language. For example, I replaced wording such as “predicts exit” with “is associated with exit” or equivalent formulations. I also revised the model/empirical boundary more generally so that the formal model is presented as identifying a mechanism, whereas the empirical section is described in descriptive, non-causal terms.

**Comment**:
I also remain unconvinced by the characterization of the algorithmic filtering dynamics as particularly “counterintuitive” or “paradoxical.” Once the mechanism is explicitly described, the result that reduced exposure to disagreeing opinions slows down exit-driven segregation appears rather intuitive. Moreover, the comparison with Ref. [32] is not entirely accurate. Pansanella et al. show that algorithmic filtering slows down segregation dynamics, whereas in the absence of such filtering echo chamber formation proceeds more rapidly, an outcome that is broadly consistent with the mechanism proposed here. While relocation across communities is not present in that model (nor in Valensise et al.), rewiring links away from disagreeing neighbors represents a closely related mechanism: rather than exiting an entire community, agents exit specific social ties in response to disagreement. In both cases, the removal of disagreeing interactions drives segregation. Although the formation of new links in those models is guided by homophily, the key point remains that algorithmic filtering dampens, rather than accelerates, segregation dynamics, reducing the perceived disagreement and inhibiting the driver of the exit behaviour.

**Response**:
Thank you. I revised this section substantially in light of your comment.

First, I removed or toned down the terms “counterintuitive” and “paradoxical” in describing the filtering result. The revised text now presents the result more neutrally, as a consequence of the interaction between exposure, dissatisfaction, and exit dynamics.

Second, I revised the comparison with Pansanella et al. to make it more accurate. The manuscript now explicitly notes that prior modeling work has found different effects of filtering depending on the underlying mechanism, and it now acknowledges that Pansanella et al. show that filtering can slow segregation by reducing disagreeing encounters. I also clarify that the present model differs in interaction structure: here segregation is driven by exit from bounded communities, whereas in that framework it operates through rewiring away from disagreeing neighbors. The revised text now presents these as related mechanisms rather than as contrasting findings.

**Comment**:
the z-standardization of the logistic regression could be better clarified, e.g., "All continuous predictors are z-standardized (mean 0, standard deviation 1), so coefficients can be interpreted as the effect of a one–standard-deviation increase in the corresponding variable on the log-odds of exit."

**Response**:
Thank you. I adopted this clarification in the empirical section. The manuscript now explicitly states that all continuous predictors are z-standardized with mean 0 and standard deviation 1, and that coefficients can be interpreted as the change in log-odds associated with a one-standard-deviation increase in the corresponding variable.

**Additional changes made in response to the overall concerns**

In addition to the point-by-point revisions above, I made several broader changes to improve the manuscript’s consistency and precision.

The title was revised to avoid the earlier implication that the model excludes all preference. It now emphasizes that echo chambers can emerge without algorithmic personalization or a preference for *homogeneity*.

The abstract was rewritten to foreground the model’s main claim in more precise terms: strong segregation can emerge without algorithmic personalization and without users actively seeking homogeneous environments, and the empirical analysis is presented as an illustration compatible with the model’s micro-level assumption.

The introduction was revised to remove statements suggesting that users in the model “prefer diversity” or “prefer mixed environments.” These are now replaced with language stating that users tolerate substantial disagreement or do not strongly prefer homogeneous environments.

In the model section, I replaced the phrase “conservative lower bound” with “minimal mechanism” and clarified that the final segregation value refers to the segregation level at the end of the simulation horizon, since the model does not necessarily reach equilibrium within the finite number of steps shown in the figures.

In the empirical section, I consistently shifted from “ideological distance” to “linguistic distance” or “semantic distance” where appropriate, in order to match the measure actually used and to avoid overstating what the centroid captures.

In the discussion, I softened several remaining claims that implied replacement rather than complementarity. The revised conclusion now states explicitly that the proposed mechanism is not a replacement for existing accounts of online homophily or algorithmic filtering, but a complementary structural pathway through which segregation can arise.

I am grateful for these comments, which helped sharpen the manuscript considerably. I hope the revised version now addresses the remaining concerns clearly and satisfactorily.
